# Schwann cells in regeneration and cancer

**DOI:** 10.3389/fphar.2025.1506552

**Published:** 2025-01-29

**Authors:** Lan Zhang, Jiale Xie, Wenyu Dai, Bing Lu, Sheng Yi

**Affiliations:** ^1^ Key Laboratory of Neuroregeneration of Jiangsu and Ministry of Education, Co-innovation Center of Neuroregeneration, NMPA Key Laboratory for Research and Evaluation of Tissue Engineering Technology Products, Nantong University, Nantong, Jiangsu, China; ^2^ Department of Clinical Biobank and Institute of Oncology, Affiliated Hospital of Nantong University, Nantong University, Nantong, Jiangsu, China

**Keywords:** Schwann cells, cell plasticity, tissue regeneration, tumorigenesis, tumor progression

## Abstract

Schwann cells are specific peripheral glial cells with remarkable plasticity following peripheral nerve injury. Injury responses stimulate c-Jun activation in Schwann cells, drive epithelial-mesenchymal transition and cellular phenotypic changes, and induce the generation of reprogrammed repair Schwann cells to orchestrate peripheral nerve regeneration process. Schwann cells and/or Schwann cell-derived molecules are commonly used as supporting cells and/or neurotrophic factors to construct Schwann cell-based tissue-engineered nerve grafts for repairing severe peripheral nerve injury with long defects. Transplantation of Schwann cells and/or Schwann cell-derived molecules also serves as a helpful approach for the treatment of other injured tissues, such as the spinal cord, skin, digit tip, and bone. Schwann cells are not only associated with tissue regeneration but also involved in tumorigenesis and tumor progression. Schwann cells are the major cellular component of neurofibromatosis type 1 and the sole cell type in neurofibromatosis type 2 and schwannomatosis. In addition, Schwann cells also function as an important player in the tumor microenvironment and aid in the growth and invasiveness of many other solid cancers. In the present review, we outline the physiological and pathological activities of Schwann cells and discuss the functional roles of Schwann cells in homeostasis, regeneration, and cancer.

## Introduction

**FIGURE 1:**
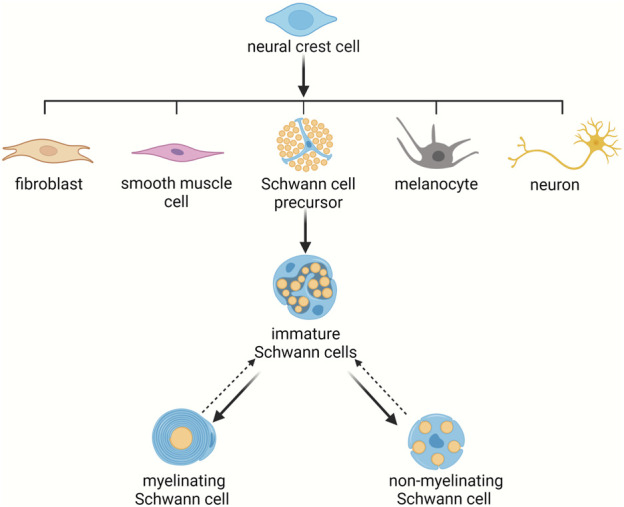
Schwann cell lineage during development. Schwann cell precursors arise from neural crest cells and differentiate to immature Schwann cells. Immature Schwann cells next give rise to mature Schwann cells, including myelinating Schwann cells and non-myelinating Schwann cells.

Schwann cells are unique glial cells of the peripheral nervous system that are derived from the neural crest. During development, neural crest cells emerge from the dorsal neural tube and differentiate into numerous types of cells, including melanocytes, fibroblasts, smooth muscle cells, neurons, and Schwann cell precursors (Jessen and Mirsky, 2005; Takahashi et al., 2013). Schwann cell precursors generate immature Schwann cells which express some common markers as neural crest cells and Schwann cell precursors, such as the neuregulin (NRG) receptors Erb-B2 receptor tyrosine kinase 2 (ErbB2), Erb-B2 receptor tyrosine kinase 3 (ErbB3), and Erb-B2 receptor tyrosine kinase 4 (ErbB4), the neurotrophin receptor p75, transcription factors SRY-box 2 (Sox2) and SRY-box 10 (Sox10), and mesenchymal marker vimentin (Aquino and Sierra, 2018). Immature Schwann cells, compared with their precursor cells, have a reduced amount of mesenchymal marker N-cadherin but high abundance of early growth response 2 (Krox20) and glial fibrillary acidic protein (GFAP) (Aquino and Sierra, 2018). Postnatally, immature Schwann cells give rise to myelinating Schwann cells that not only express pan-Schwann markers ErbB3 and S100 but also express myelin proteins such as myelin basic protein (Mbp), myelin protein zero (Mpz), and proteolipid protein 1 (Plp1) as well as non-myelinating Schwann cells that express p75, glutamate-ammonia ligase (Glul), apolipoprotein D (Apod), SPARC related modular calcium binding 2 (Smoc2), and fatty acid binding protein 7 (Fabp7) (Avraham et al., 2020; Wolbert et al., 2020). Myelinating Schwann cells spirally wrap large diameter axons, form compact myelin lamellae around axons, produce mature myelin sheaths, and thus contribute to the rapid conduction of nerve impulses. Non-myelinating Schwann cells occupy a larger proportion than myelin forming Schwann cells. Non-myelinating Schwann cells can be further divided into Remak Schwann cells, terminal Schwann cells at neuromuscular junctions, and Schwann cells in sensory corpuscles (Griffin and Thompson, 2008). Remak Schwann cells, although do not form compact myelin sheaths, also ensheath axon lengths. Different from myelinating Schwann cells that ensheath axons at a 1:1 ratio, each Remak Schwann cell ensheathes multiple small-caliber axons (Figure 1) (Armati and Mathey, 2013; Harty and Monk, 2017).

## Injury responses of Schwann cells

**FIGURE 2:**
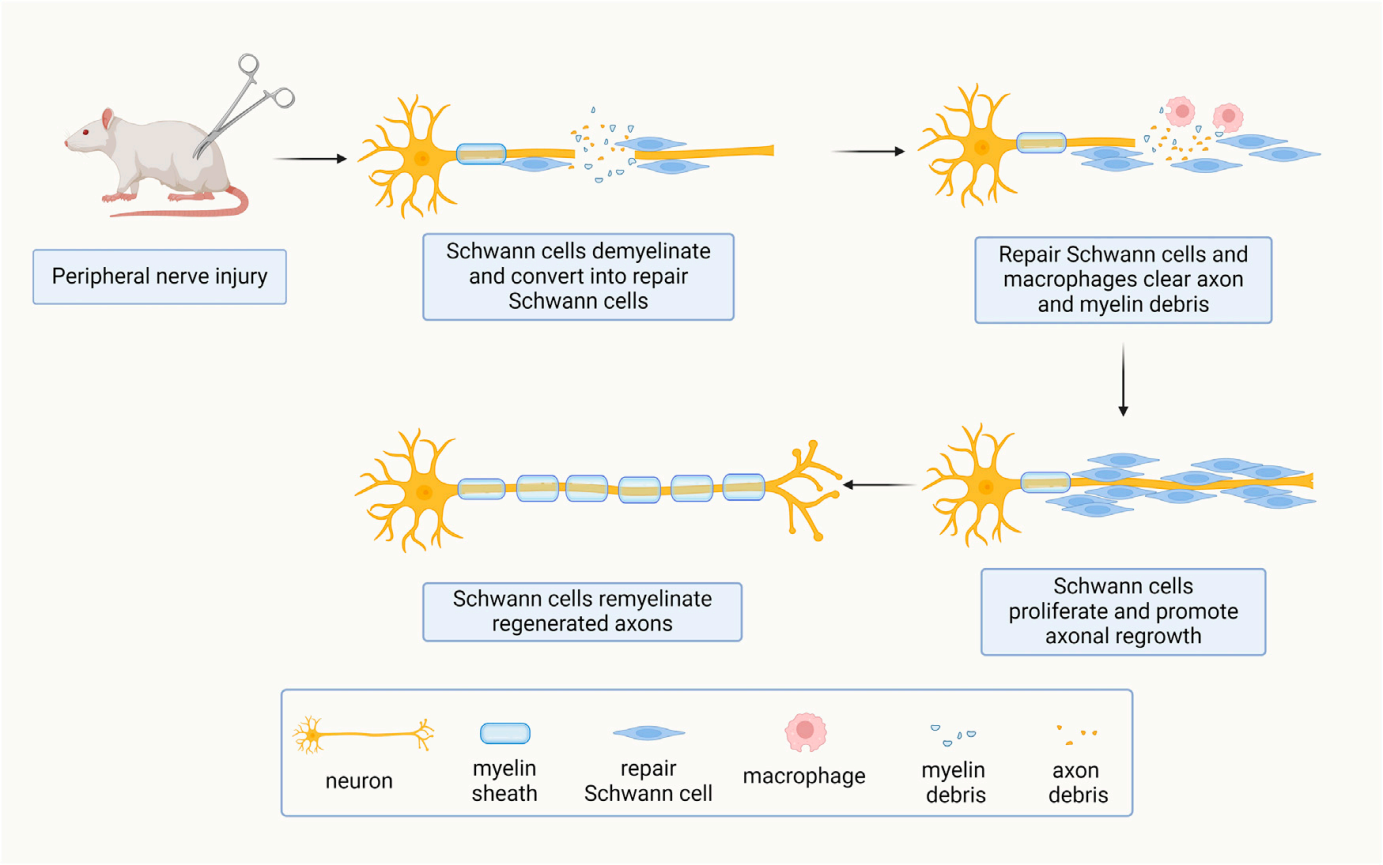
Schwann cells respond to peripheral nerve injury. Upon peripheral nerve injury, mature axon-bundling Schwann cells switch to a repair phenotype essential for nerve regeneration.

Mature Schwann cells are not only essential for the maintenance of normal nerve integrity and function, but are also critical for the regeneration process. After peripheral nerve injury, axons and their surrounding Schwann cells are disrupted. The distal nerve stumps, which are disconnected from neuronal cell bodies undergo Wallerian degeneration. During this process, both myelinating Schwann cells and non-myelinating Remak Schwann cells disassemble from their surrounding axons, undergo radical phenotypic and genetic changes, and transform into dedifferentiated and activated repair Schwann cells (Figure 2) (Jessen and Mirsky, 2016).

Sequencing analyses of the distal sciatic nerve stumps of adult mice and contralateral uninjured sciatic nerves reveal the downregulation of a large number of myelin-associated genes, including Mbp, Mpz, peripheral myelin protein 22 (Pmp22), periaxin (Prx), dystrophin related protein 2 (Drp2), and cadherin 1 (Cdh1) and the upregulation of a large number of repair-associated genes, including nerve growth factor receptor (Ngfr), galectin 3 (Lgals3), activating transcription factor (Atf3), sonic hedgehog signaling molecule (Shh), and glial cell derived neurotrophic factor (Gdnf) at 7 days after sciatic nerve cut as well as an earlier upregulation of transcription factor c-Jun coding gene within the first 24 h after nerve cut (Arthur-Farraj et al., 2017). The fast increase of c-Jun is consistent with a previous observation that c-Jun regulates the expressions of many trophic factors and drives Schwann cell reprogramming during Wallerian degeneration (Arthur-Farraj et al., 2012). Upregulated c-Jun is reported to be a downstream cascade of the ERK signaling pathway. Activated ERK signaling induces demyelination in the absence of nerve injury and mediates Schwann cell dedifferentiation after nerve injury (Napoli et al., 2012). Notch, another Schwann cell demyelination-initiating factor, is also identified to be regulated by activated ERK signaling (Napoli et al., 2012; Woodhoo et al., 2009). Other factors, including elevated expression of transcription factor Sox2 and reduced expression of transcription factor Krox-20 in the injured sites after peripheral nerve injury, also contribute to the activation and dedifferentiation of Schwann cells (Zhang et al., 2023).

Moreover, nerve injury-induced differentially expressed genes in the distal nerve stumps are found to be highly involved in epithelial-mesenchymal transition (EMT), a critical cellular programme for cellular plasticity and wound healing (Arthur-Farraj et al., 2017; Dongre and Weinberg, 2019). Increased N-cadherin expression in repair Schwann cells, a hallmark of activated EMT, is just opposite to decreased N-cadherin in Schwann cell lineage, reflecting a developmental regression and the high cellular plasticity of repair Schwann cells. The transcriptomes of purified Schwann cells collected from the distal nerve stumps and the nerve bridge connecting the severed nerve stumps show that the EMT transition from intact Schwann cells to distal Schwann cells is more enriched in bridge Schwann cells (Clements et al., 2017). Transforming growth factor beta (TGF-β), the master regulator of the EMT process, is profoundly expressed in bridge Schwann cells. Highly expressed TGF-β drives Schwann cells in the wound microenvironment to mesenchymal-like cells (Clements et al., 2017). Altered extracellular matrix, a feature closely associated with TGF-β signaling and EMT (Peng et al., 2022), is also observed in Schwann cells at the injured site (Brosius Lutz et al., 2022).

Injury-induced Schwann cells exhibit certain similarity as transcription factors and/or small molecules-driven direct cellular reprogramming and are thus referred as adaptive cellular reprogramming (Jessen and Arthur-Farraj, 2019; Wang et al., 2021). These physiologically reprogrammed Schwann cells recruit macrophages to initiate inflammatory responses, engulf axon and myelin debris to clear a regeneration path, secrete neurotrophic factors to support neuronal survival and axonal regrowth, build a favorable microenvironment for axonal regeneration, and migrate across the wound to guide the directional elongation of regenerated axons towards the distal nerve stumps. At a later stage of nerve regeneration, activated Schwann cells contact with regenerated axons and then re-differentiate and ensheath axons again to facilitate the functional recovery of injured peripheral nerves.

## Applications of Schwann cells in peripheral nerve regeneration

**FIGURE 3:**
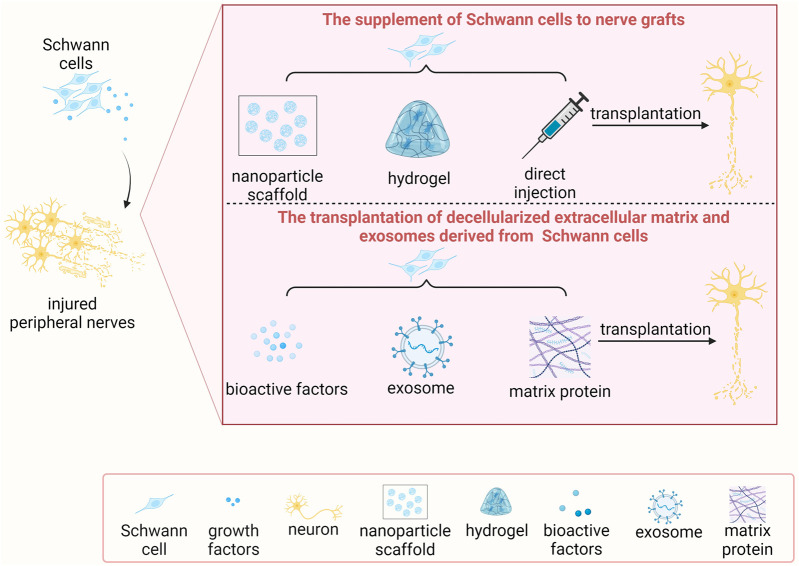
The transplantation of Schwann cells and/or Schwann cell-based molecules for treating peripheral nerve injury.

The self-repairing ability of mammalian tissues is relatively uncommon and can only be seen in a few tissues and organs, including liver and skin. With the remarkable adaptive reprogramming feature of Schwann cells, the peripheral nervous system exhibits distinct characterizes as the central nervous system and possesses an intrinsic regenerative capacity. However, the regeneration speed of injured peripheral nerves is slow (approximately 1–3 mm per day). After severe peripheral nerve injury, especially injury with long nerve gap, relying on the adaptive reprogramming of Schwann cells and spontaneous regeneration of injured peripheral nerves alone is insufficient to avert the atrophy of denervated targets prior to successful nerve regeneration and target tissue reinnervation (Yi et al., 2019). The supplementation of additional Schwann cells to the injured sites thus serves as an important therapeutic avenue for the treatment of severe peripheral nerve injury. Particularly, using mitomycin C-pretreated acellular autografts to bridge transected sciatic nerves, compared with cellular autografts, debris is cleaned up at a slower speed, very few neurites regenerate into the nerve grafts, and remyelination is not established (Hall, 1986). These findings directly illuminate the essential roles and application prospects of Schwann cells in peripheral nerve regeneration (Figure 3).

### Application of Schwann cell-based nerve grafts

Schwann cells can be suspended and encapsulated in injectable hydrogels and directly applied to the injured sites (Rao et al., 2022). For a more effective and functional delivery of Schwann cells, Schwann cells are commonly introduced into nerve grafts, preferably nerve conduits containing internal axon guidance channels, as supporting cells to treat severe peripheral nerve injury (Errante et al., 2022; Yi et al., 2020). Tubulization of long-distance peripheral nerve defects, such as 6 mm in mice, 10–15 mm in rats, and 30 mm in rabbits and primates, with plain nerve grafts generally leads to regeneration failures (Udina et al., 2004; Vallejo et al., 2022). Supplementing Schwann cells into natural and/or artificial nerve grafts offers hope to break the upper limit of peripheral nerve regeneration.

In a white New Zealand rabbit model, autogenous venous nerve conduits filled with 1 × 10^6^/mL Schwann cells and Matrigel successfully repair 6-cm-long peroneal nerve gaps. Compared with Matrigel-only filled nerve conduits, the presence of Schwann cells supports axon elongation throughout the nerve conduits and increases the interactions between Schwann cells and axons. The histoarchitectures and cytoarchitectures of Schwann cell/Matrigel-filled autogenous venous nerve conduits seem to be identical to normal rabbit peroneal nerves at 4 months after nerve bridging (Strauch et al., 2001).

Similarly, 1.5 × 10^5^ Schwann cells are suspended in Matrigel and then seeded in poly (L-lactide-co-ε-caprolactone) nerve grafts to bridge 6-mm-long sciatic nerve gaps in Swiss OF1 mice (Rodríguez et al., 2000). Mice transplanted with autologous Schwann cells filling nerve grafts have a larger number of regenerated fibers, higher amplitudes of compound muscle action potential, and higher pinprick scores than mice transplanted with nerve grafts containing Matrigel alone. Notably, the functional roles of transplanted isologous and syngeneic Schwann cells in peripheral nerve regeneration are not as good as autologous Schwann cells, indicating the influence of immunogenicity (Rodríguez et al., 2000). A subsequent study further demonstrates that the administration of immunosuppressant FK506 speeds up the onset of reinnervation and achieves improved functional recovery (Udina et al., 2004).

Using absorbable collagen conduits and/or 3D collagen matrix nerve conduits filled with 2 × 10^5^/μL autologous Schwann cells to repair 13-mm-long sciatic nerve gaps in Fischer rats results in long-term survival of implanted Schwann cells and increased number of myelinated axons throughout the entire nerve conduit (Berrocal et al., 2013; Burks et al., 2021). Chitosan conduits combined with collagen and laminin- or fibronectin-aligned matrices as well as 7.5 × 10^5^ Schwann cells are used to bridge even longer nerve gaps, that is 15-mm-long sciatic nerve defects in Wistar rats (Gonzalez-Perez et al., 2018). The introduction of Schwann cells results in full regeneration in terms of electrophysiological and functional recovery parameters and leads to better effects than mesenchymal stem cells, a type of commonly used stem cells in tissue engineering and regenerative medicine (Gonzalez-Perez et al., 2018). Schwann cell-based nerve grafts are effectively used to bridge even longer peripheral nerve gaps, for instance, 17-mm-long sciatic nerve defects in Sprague Dawley rats (Ma et al., 2020) and 20-mm-long sciatic nerve defects in Lewis rats (Hoben et al., 2015).

The development of the understanding of regulating factors of Schwann cell behavior allows the addition of genetically modified Schwann cells and/or chemical-treated Schwann cells to neural tissue engineered grafts and may further facilitate peripheral nerve regeneration. For instance, Schwann cells transfected with a recombinant retrovirus vector containing the glial cell line-derived neurotrophic factor (GDNF) gene, a gene that encodes a well-known growth factor with neurotrophic functions, support motor neuron survival *in vitro*. When filled in a silicone tube to repair 10-mm long defects in Wistar rats, GDNF-modified Schwann cells enhance myelin sheath formation as well as nerve conductance, achieving a better outcome than normal Schwann cells (Li et al., 2006). Similarly, the transplantation of poly (lactic-co-glycolic acid) biodegradable conduits combined with nanoparticles carrying Schwann cells transfected with another neurotrophic factor, neurotrophin-3 (NT-3), accelerates axonal regrowth, promotes myelin regeneration, and benefits functional recovery of injured nerves (Zong et al., 2013).

Transduction of Schwann cells with a lentiviral vector encoding c-Jun, the essential regulator of Schwann cell reprogramming, upregulates the secretion of numerous growth factors with neurotrophic activities, such as GDNF, nerve growth factor (NGF), brain-derived neurotrophic factor (BDNF), and artemin (Huang et al., 2015). Application of c-Jun-modified Schwann cells to poly (ε-caprolactone) nerve conduits rises the survival rate of sensory neurons, speeds up the elongation of injured axons, alleviates target muscle atrophy, enlarges the compound muscle action potential, and increases sciatic function index, an indicator of nerve function (Huang et al., 2019). Moreover, by using a tetracycline-regulatable Tet-On system, the expression levels of c-Jun in Schwann cells can be strictly regulated. Hence, the subsequent secretion of c-Jun-regulated growth factors can be controlled to realize optimal release kinetics of neurotrophic factors (Huang et al., 2019).

Clinical trials have evaluated the beneficial roles of Schwann cell-based treatments for peripheral nerve injury. Levi et al. isolated human autologous Schwann cells from dissected sural nerve and sciatic nerve biopsy, expanded Schwann cells with heregulin β1 and forskolin to obtain a larger population of 2.9 × 10^7^ cells, and combined with an FDA-approved collagen matrix Duragen^®^ to bridge a 7.5-cm-long sciatic nerve gap in a female patient with sustained lacerating wound to right posterior thigh (Levi et al., 2016). A 24-month clinical follow-up showed that after nerve bridging, the patient experienced proximal sensory recovery, definitive motor recovery, and certain pain relief while does not form tumors, demonstrating the effectiveness and safety of this Schwann cell-based therapy (Levi et al., 2016). Another clinical study also harvested autologous Schwann cells from traumatized sciatic nerves and/or sural nerves and used a Duragen Secure Dural Regeneration Matrix supplied with 2.88 × 10^7^ Schwann cells to repair a 7.5-cm-long sciatic nerve defect in a female patient with lacerating injury as well as a Duragen Secure Dural Regeneration Matrix supplied with 1.1 × 10^8^ Schwann cells to repair a 5-cm-long tibial nerve defect in a female patient with a gunshot wound. In both cases, implanted Schwann cell-based biomaterials did not induce postoperative complications while patients gained certain sensory functional recovery and complete motor function recovery (Gersey et al., 2017). A recent study, by evaluating Schwann cell culture manufacturing data from human patients, offers a reliable and productive method for the isolation, enzymatic dissociation, and expansion of high-quality Schwann cells from harvested autologous nerves and thus allows the potential for the clinical application of autologous Schwann cells in treating neurological disorders (Khan et al., 2022).

To overcome the limitation of the lack of the clinically available Schwann cells, stem cell-derived Schwann-like cells have been used as regenerative therapy. Schwann-like cell spheroids generated from adipose-derived stem cells have been successfully applied in a mouse sciatic nerve injury model, exerting good therapeutic effect on structural and motor function recovery (Chen et al., 2022).

### Application of Schwann cell-derived molecules

It is worth noting that no matter how the method of autologous Schwann cell culture develops, the direct application of autologous Schwann cells needs an added invasive surgery to collect autologous nerve grafts, and therefore, induces autologous nerve injury and secondary deformities. Furthermore, the acquisition of adequate amount of Schwann cells is time consuming and may hamper patients to receive timely treatment. The usage of allogeneic and/or xenogeneic Schwann cells evades those inherent disadvantages of autologous Schwann cells. However, allogeneic and/or xenogeneic Schwann cells may induce immune rejection due to high antigenicity and thus negatively influence the success of cellular therapy. Schwann cell-derived molecules, especially extracellular matrices and exosomes, have positive effects on nerve regeneration while obtain the characteristics of low immunogenicity and low toxicity. Therefore, compared with the direct transplantation of allogeneic and/or xenogeneic Schwann cells, the transplantation of decellularized extracellular matrix and exosomes derived from allogeneic and/or xenogeneic Schwann cells is more promising and may expand the usage of Schwann cells in clinical practice.

Decellularized extracellular matrix retains the original structure and molecular components and offers important biophysical and biochemical information during tissue repair (Yi et al., 2017). Moreover, decellularized extracellular matrix derived from allogeneic and/or xenogeneic Schwann cells does not elicit acute or chronic inflammatory responses after implantation. It is observed that following the decellularization of rat Schwann cell-derived extracellular matrix, cellular DNA has been removed. Fibronectin and laminin, two major extracellular matrix components, as well as the original structure of the extracellular matrix are reserved. The joint use of allogeneic Schwann cell-derived extracellular matrix and chitosan/silk fibroin nerve conduits encourages the regeneration and remyelination of injured nerves in Sprague Dawley rats with no observed adverse effects, resulting in better treatment outcomes than acellular nerve tissue-derived extracellular matrix scaffolds (Gu et al., 2014).

Exosomes, as cell-derived effective therapeutic extracellular vesicles, are considered as a promising element in cell-free regenerative medicine (Kimiz-Gebologlu and Oncel, 2022). Schwann cell-derived exosomes can be specifically internalized by axons and then stimulate axon elongation and growth cone extension (Lopez-Verrilli et al., 2013). When injected to crushed sciatic nerves in Sprague Dawley rats, exosomes derived from allogeneic Schwann cells are observed to be internalized by neuronal axons. Injured axons regrow to a two times longer distance than vehicle-injected axons at 4 days after surgery (Lopez-Verrilli et al., 2013). Remarkably, the regeneration-promoting ability of exosomes largely depends on cellular status. Consistent with the beneficial role of repair Schwann cells in the process of peripheral nerve regeneration, exosomes secreted from repair Schwann cells are more propitious to neurite outgrowth as compared with exosomes secreted from pharmacologically modulated differentiated Schwann cells (López-Leal et al., 2020). In addition to repair Schwann cells, exosomes secreted from Schwann cells that are treated with superparamagnetic iron oxide nanoparticles-containing cell culture medium and subjected to the magnetic field also have positive effects on neurite outgrowth (Xia et al., 2020).

## Applications of Schwann cells in tissue regeneration

**FIGURE 4:**
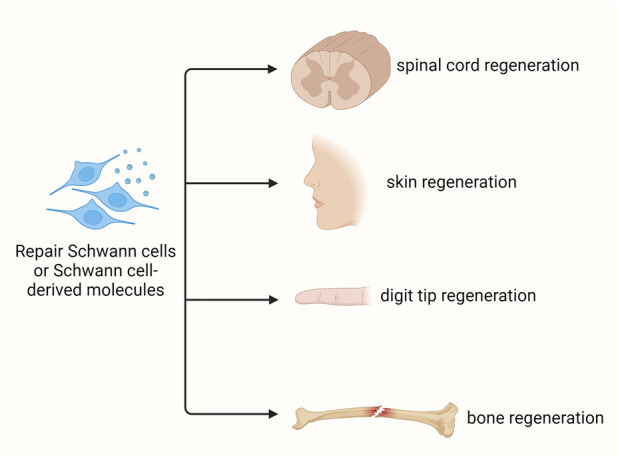
Schwann cell-based regenerative medicine. Schwann cells and/or Schwann cell-based molecules benefit the regeneration of various tissues, including spinal cord, skin, digit tip, and bone.

In addition to injured peripheral nerves, Schwann cells have been used as promising donor cells for the regeneration of numerous types of other tissues and organs, such as spinal cord, skin, digit tip, and bone (Figure 4).

### Spinal cord regeneration

Considering the advantageous role of peripheral nerve grafting in central nerve repair, Schwann cells, as a major cellular composition of peripheral nerves, have been used to treat central nerve injury for a long period (Hall et al., 2022). A transplantation of 2 × 10^6^ cultured adult rat Schwann cells to adult Fischer rats with moderate contusion at the thoracic (T9) spinal cord reduces localized lesion cavitation, supports the elongation of corticospinal, propriospinal, and brainstem nerve fibers, and advances hindlimb locomotor recovery (Takami et al., 2002). The addition of immunosuppression increases the survival of implanted allogenic Schwann cells and further improves regenerative outcomes (Hill et al., 2006). The incorporation of additional strategies, such as bioactive materials, neuroprotective drugs, and exogenous or gene modified neurotrophic factors, ulteriorly supports the growth of axons into Schwann cell implants (Bunge, 2016; Wiliams and Bunge, 2012). For instance, after the transplantation of Schwann cell-seeded piezoelectric polyvinylidene fluoride trifluoroethylene conduits, the formation of blood vessels and the presence of regenerated sensory axons are detected in rats with complete spinal cord transection (Lee et al., 2017). Therapeutically, grafting manufactured sural nerve-isolated autologous Schwann cells in human patients with spinal cord injury does not induce surgical or neurological concerns, indicating the safety and feasibility of Schwann cell-based therapy (Anderson et al., 2017; Gant et al., 2022; Guest et al., 2013). Certain level of motor, sensory, and autonomic functional recovery are observed in patients with spinal cord injury during a 5-year follow-up after autologous Schwann cells transplantation (Zhou et al., 2012).

Still, the difficulty of producing liberal quantity of clinical grade autologous Schwann cells limits the translational application of Schwann cells in treating spinal cord injury. To avoid immune-mediated rejection, emerging studies have applied Schwann cell-derived molecules, such as exosomes, to the field of central nerve regeneration. It has been demonstrated that the injection of Schwann cell-derived exosomes to rodents decreases astrocyte-secreted regeneration inhibitory factor chondroitin sulfate proteoglycans by activating the NF-κB/PI3K signaling, mediates the M1 to M2 phenotypic switch of macrophages by activating the SOCS3/STAT3 signaling, elicits the autophagy of neurons by inhibiting the Akt/mTOR signaling, and mediates electrophysiological and motor function recovery (Pan et al., 2021; Pan et al., 2022; Ren et al., 2023; Zhu et al., 2023).

### Skin regeneration

Mammalian skin is capable of self-regeneration due to the considerable plasticity of various cell types in the skin wound, including keratinocytes, dermal fibroblasts, mesenchymal stem cells, and immune cells (Shaw and Martin, 2016). Schwann cells are also functionally important in the skin wound healing process as injury-activated Schwann cells express multiple growth factors and assist skin wound closure (Johnston et al., 2013; Parfejevs et al., 2018). Interestingly, injury-activated Schwann cells endorse myofibroblast formation by paracrine modulation of TGF-β signaling, a signaling pathway that mediates the formation of repair Schwann cells (Parfejevs et al., 2018). Hence, a generated positive feedback loop may further facilitate skin regeneration. On the other hand, moderated injury responses and delayed Schwann cell dedifferentiation impair skin wound healing (Zhou et al., 2022).

### Digit tip regeneration

Similar to skin, the distal digit does not lose its regeneration ability during aging and is able to self-repair at maturity. Following a comprehensive regrowth and appropriate patterning of nail, bone, nerve, vessel, and connective tissue, injured distal digit regains its original structure and function (Carr and Johnston, 2017). The patterning defects of regenerated digit tip in the absence of peripheral nerves link Schwann cells with digit tip regeneration (Rinkevich et al., 2014). The fact that ablation of dedifferentiated Schwann cells suppresses digit tip regeneration while transplantation of exogenous Schwann cells isolated from the neonatal rat sciatic nerves rescues tissue regeneration straightforwardly illuminates the importance of Schwann cells in digit tip regeneration. Mechanical studies show that dedifferentiated Schwann cells secrete paracrine factors oncostatin M and platelet-derived growth factor AA and thus mediate nail and bone regeneration (Johnston et al., 2016).

### Bone regeneration

Schwann cells also contribute to the regeneration of bones localized in other positions besides the digit tip. The organogenesis and growth of tooth is closely associated with mesenchymal stem cells derived from Schwann cells (Kaukua et al., 2014). In an alveolar injury model, after the transplantation of teased sciatic nerves to tooth extraction sockets of C57BL/6 mice with tooth extraction, Schwann cells move towards the alveolar defect and dedifferentiate into repair Schwann cells. Dedifferentiated Schwann cells boost alveolar skeletal stem cell proliferation and enhance alveolar bone regeneration by providing trophic supports (Zhang et al., 2023). In a femur defect model, bridging of 6-mm-long bone defects in Sprague Dawley rats with engineered bone grafts seeded with co-cultured Schwann cells and induced endothelial cells, as compared with engineered bone grafts seeded with induced endothelial cells alone, has better effects on scaffold prevascularization and osteogenesis (Zhang et al., 2021). Similar to Schwann cells, the addition of Schwann cell-derived exosomes also induces angiogenesis and osteogenesis (Hao et al., 2023). A larger abundance of M2 macrophage-related genes is also detected after the application of Schwann cell-derived exosomes, showing that Schwann cell-derived exosomes facilitate a M1 to M2 macrophage phenotype switch and contributes to the regulation of immune responses (Hao et al., 2023). Following the addition of Schwann cell-derived exosomes to bone marrow stromal cells, the proliferation and migration as well as the formation of an osteoblast-like cell morphology in bone marrow stromal cells are stimulated. *In vivo* observations show that when combined with 3D porous Ti6Al4V scaffolds to repair bone defect in New Zealand white rabbits, Schwann cell-derived exosomes enhance the formation of new bones in a dose-dependent manner (Wu et al., 2020).

## Functions of Schwann cells in tumorigenesis and tumor progression

**FIGURE 5:**
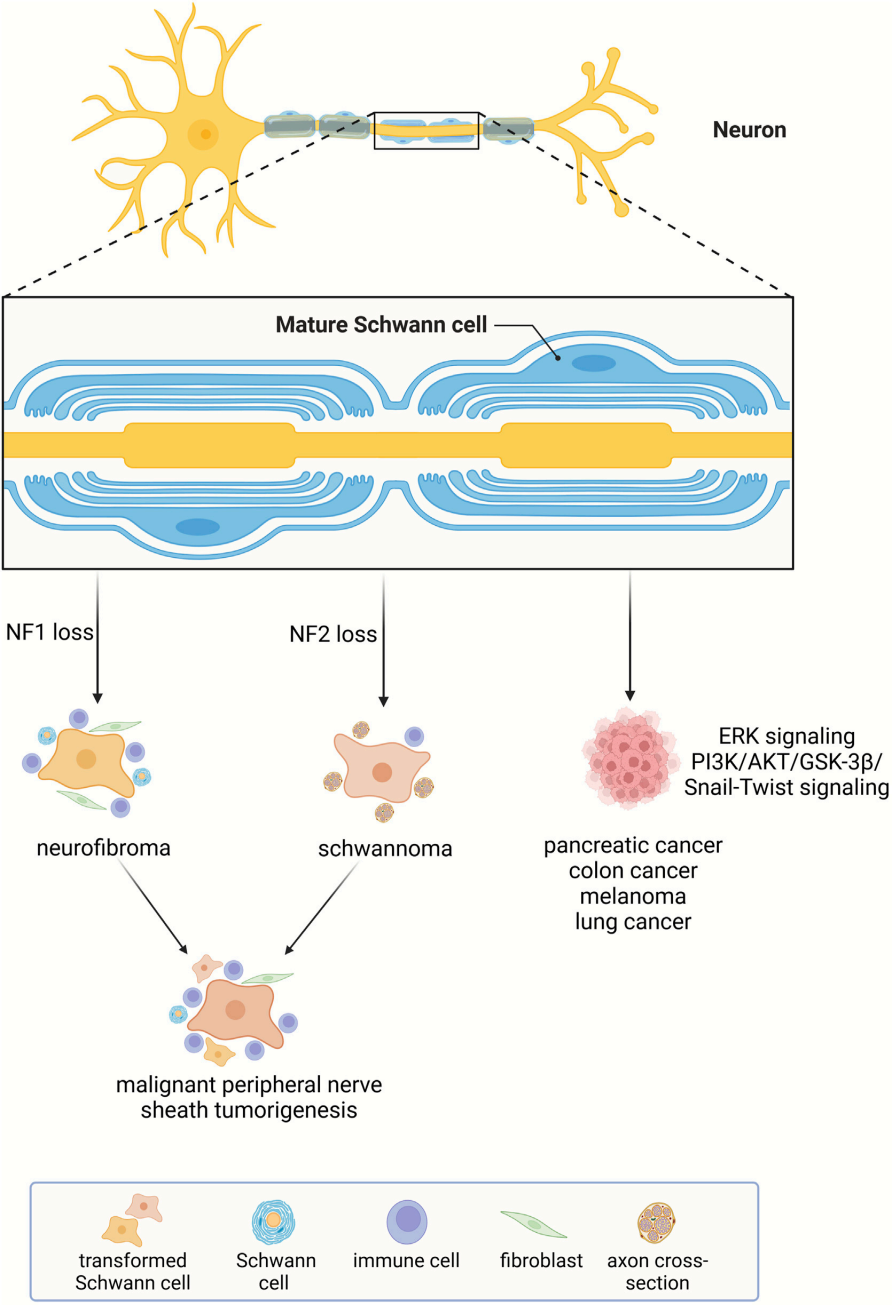
Schwann cell-related cancers. Schwann cells participate in the formation of neurofibromatosis and affect the growth and invasiveness of many other types of cancers.

Tissue regeneration and tumorigenesis own many common cellular and molecular bases (Charni et al., 2017; Oviedo and Beane, 2009). Schwann cells have been closely linked with the regeneration of injured peripheral nerves as well as the initiation of neurofibromatosis, that is the tumorigenesis of peripheral nerves. The wide anatomical distribution of Schwann cells and the regenerative capacity of Schwann cells to surrounding tissues, such as skin and digit tip imply the potential participation of Schwann cells in the development and progression of cancers in other tissues and organs besides peripheral nerves. Many recent findings unmask the interaction between Schwann cells and a series of solid tumors. Herein, the involvement of Schwann cells in cancers is introduced (Figure 5).

### Neurofibromatosis

Neurofibromatosis is a group of nerve-associated cutaneous and/or internal tumors with three major types, i.e., neurofibromatosis type 1, neurofibromatosis type 2, and schwannomatosis (McClatchey, 2007). Neurofibromatosis type 1 is caused by familiar and/or new mutations of tumor suppressor gene neurofibromin 1 (NF1). It is a common genetic disorder with a high incidence of one per 3,500 individuals (Myers, 1991). Neurofibroma is a classic symptom in patients with neurofibromatosis type 1. In these patients, cell populations in the peripheral nerves, including Schwann cells, axonal processes, perineurial cells, fibroblasts, and mast cells, co-constitute benign neurofibroma (Zhu et al., 2002). Additional mutations in other tumor suppressor genes and aberrant activation of growth factor signaling induce the progression of neurofibroma to extremely metastatic malignant peripheral nerve sheath tumors (Carroll, 2016).

Incidence of neurofibromatosis type 2 and schwannomatosis is much lower than neurofibromatosis type 1. Tumors in these patients generally do not progress to malignancy (McClatchey, 2007). Unlike the complex cellular compositions of neurofibromas in neurofibromatosis type 1 patients, tumors in neurofibromatosis type 2 and schwannomatosis patients are composed solely of Schwann cells and are therefore named schwannoma (Carroll, 2012). In neurofibromatosis type 2 patients, the schwannoma often develops on or around the vestibular branches of auditory nerves while in schwannomatosis patients, the schwannoma does not occur on the vestibular nerve (McClatchey, 2007). In addition, neurofibromatosis type 2 and schwannomatosis patients have diverse genetic backgrounds. Neurofibromatosis type 2 is caused by mutation of neurofibromin 2 (NF2) gene mutation, a tumor suppressor gene with inhibitory roles in cell proliferation while the inherited loss of NF2 is not the cause of schwannomatosis (Kehrer-Sawatzki et al., 2017; MacCollin et al., 2003).

Despite the genetic and phenotypic diversities of neurofibromatosis type 1, neurofibromatosis type 2, and schwannomatosis, a common cellular basis of all these forms of neurofibromatosis is Schwann cells. As the cell-of-origin for neurofibromatosis, the activity of Schwann cell is highly related to the tumorigenesis of peripheral nerves. For instance, Schwann cell reprogramming driven by TAZ/YAP signaling mediates the pathogenesis of malignant peripheral nerve sheath tumors (Wu et al., 2018). Regulating the cellular behavior of Schwann cells thus serves as a critical therapeutic avenue for the treatment of neurofibromatosis.

### Cancer progression

Recent studies demonstrate that besides neurofibromatosis, Schwann cells are closely associated with other types of tumors, especially tumors with high metastasis rates.

Pancreatic ductal adenocarcinoma is a notoriously aggressive and lethal cancer with a high metastasis rate. Pancreatic cancer cells are capable of invading the surrounding nerves through a process named perineural invasion and spreading throughout the body (Bapat et al., 2011). In a mouse model of pancreatic ductal adenocarcinoma, capsaicin injection-induced denervation significantly suppresses perineural invasion and slows tumorigenesis and cancer progression, demonstrating the participation of nerves in this aggressive cancer (Saloman et al., 2016). Reprogrammed Schwann cells are found to be closely associated with cancer cells in pancreatic histological section with perineural invasion and positively correlated with pancreatic cancer invasion and diminished survival (Deborde et al., 2022; Deborde et al., 2016). Schwann cells secrete soluble factors to elevate the invasiveness and migratory capacity of pancreatic adenocarcinoma cells by inducing the EMT process and driving tumor cells as well as cancer-associated fibroblasts to switch to more malignant subtypes (García-Reyes et al., 2023; Xue et al., 2023). In the tumor microenvironment, pancreatic adenocarcinoma cells provoke c-Jun activation in Schwann cells and mediate Schwann cell reprogramming, which may further lead to pancreatic cancer progression (Deborde et al., 2022).

An investigation of the movement of Schwann cells that are co-cultured with pancreatic cancer cells and colon cancer cells demonstrates that these cancer cells secrete chemoattractant for Schwann cells and drive the targeted growth of Schwann cells towards cancer cells instead of benign cells (Demir et al., 2014). These findings provide molecular basis for the observed enrichment of Schwann cells in pancreatic cancer and colon cancer. Further molecular investigations reveal that colon cancer cells release exosomal miR-21-5p, which suppresses the expression of Von Hippel-Lindau tumor suppressor (VHL), prevents the degradation of hypoxia inducible factor 1 subunit alpha (HIF-1α) and correspondingly, increases the transcription of NGF in Schwann cells as well as the secretion of NGF from Schwann cells. Next, Schwann cell-secreted NGF motivates the ERK signaling pathway in colon cancer cells, which augments the proliferation and migration of colon cancer cells and further increases the expression of exosomal miR-21-5p (Han et al., 2022). Conditioned medium from Schwann cells, via increasing interleukin-8 expression and modulating the tumor microenvironment, promotes the migration and invasion of colorectal cancer cells (Frick, 1987).

The crosstalk between cancer cells and Schwann cells as well as the reprogramming of Schwann cells from a mature phenotype to a repair-like phenotype is also observed in melanoma, a malignant tumor of melanocytes with a high metastasis rate, intense invasiveness, and poor prognosis (Shurin et al., 2019). Activated Schwann cells regulate the immune microenvironment, suppress anti-tumor T cells, and stimulate melanoma enlargement while inhibition of Schwann cell activation slows tumor growth rate (Kruglov et al., 2023; Shurin et al., 2019). The bidirectional cell-cell interactions between Schwann cells and melanoma cells cooperatively contribute to melanoma growth and may likely accelerate the metastasis of melanoma.

The metastasis of lung cancer can also be raised extremely in the presence of Schwann cells. Schwann cells, through secreting C-X-C motif chemokine ligand 5 (CXCL5), activate the PI3K/AKT/GSK-3β/Snail-Twist signaling pathway in lung cancer cells, mediates EMT, and increases the motility, invasiveness, and metastatic potential of lung cancer cells (Zhou et al., 2018). In small-cell lung cancer, an aggressive neuroendocrine lung cancer subtype with high malignancy and poor prognosis, tumor-associated Schwann cells display repair-like phenotype and augment the growth and invasion of small-cell lung cancer (Cao et al., 2022).

Schwann cells may participate in other cancers, except for pancreatic cancer, colon cancer, melanoma, and lung cancer, as denervation has been demonstrated to be effective in inhibiting the progression of prostate cancer (Magnon et al., 2013), tongue cancer (Raju et al., 2007; Raju et al., 2009), and gastric cancer (Polli-Lopes et al., 2003; Zhao et al., 2014). In the tumor microenvironment, Schwann cells interact with other main components and trigger nerve-cancer cell communication (Xue et al., 2023). Along with a deeper understanding of the existence of Schwann cells in the tumor microenvironment and the contribution of Schwann cells to cancer cell spreading (Zahalka and Frenette, 2020), targeting tumor-associated Schwann cells proposes a new dawn for the prevention and therapeutic treatment of cancer diseases. For instance, considering that c-Jun mediates Schwann cell activation, c-Jun N-terminal kinase inhibitor SP600125 may be helpful for suppressing Schwann cell activity and inhibiting cancer progression. Treatments that suppress schwannoma cell growth, such as c-Met inhibitor cabozantinib, the Src kinase inhibitors dasatinib and saracatinib, and celastrol, may also serve as promising therapeutics (Fuse et al., 2017; Kim et al., 2022).

It is worth noting that cancer progression shares many common biological features with the regeneration process. Cells with promoting roles in wound healing may function as essential players in tumorigenesis (Li et al., 2019). Schwann cells may exhibit similar roles in cancer progression as in tissue remodeling and organ regeneration. Hence, when applying Schwann cell-based therapy for regenerative medicine, the biological roles of Schwann cells in cancer progression should be considered. On the other hand, the current understanding of the effects of Schwann cells on regeneration may contribute to the comprehension of Schwann cells in cancer growth, progression, and therapy.

## Conclusion

Schwann cells, as unique and major peripheral glial cells, execute essential axon surrounding and supporting roles under physiological conditions. In response to peripheral nerve injury, Schwann cells can be reprogrammed to a repair phenotype to orchestrate tissue remodeling. Hence, Schwann cells have been used as important supporting cells in the field of regenerative medicine. Schwann cells, especially hyperactivated Schwann cells, on the other hand, may be important niche components of the tumor microenvironment and influence tumor progression. A comprehensive understanding of the biological activities of Schwann cells and the mechanisms underlying Schwann cell reprogramming is likely to aid in the regeneration of injured tissues and organs as well as the prevention of cancer development.
